# The Impact of an Educational Project on Cancer-Related Knowledge and Awareness Among High School Students

**DOI:** 10.1007/s13187-025-02604-3

**Published:** 2025-03-18

**Authors:** Michał P. Budzik, Marta Fudalej, Dominika Mękal, Anna M. Badowska-Kozakiewicz

**Affiliations:** Department of Oncology Propaedeutics, Medical University of Warsaw, Erazma Ciołka 27, 01-445 Warsaw, Poland

**Keywords:** Cancer prevention, Cancer education, Cancer risk factors, Health education, High school

## Abstract

Cancer is the second leading cause of death globally, making it a significant global health issue. Approximately half of cancer cases are linked solely to behavioral habits. Lifestyle changes made at a young age are easier to adopt and have lasting effects on future health, making early awareness about cancer essential. The “OncoAcademy: The Key to Health” project, aimed at high school Polish students, has been created to promote a positive health attitude and increase awareness among young people about cancer and methods of its prevention. From September 2023 to November 2024, we conducted educational sessions for 3000 high school students and assessed their cancer prevention knowledge before and after the project. Results revealed that students’ level of knowledge before classes was moderate (*M* = 51.76%; SD = 13.63%). On average, students correctly answered 13 of 26 questions. After classes, the level of knowledge was assessed as moderately good (*M* = 62.47%; SD = 14.48%). On average, students correctly answered 16 out of 26 questions. The analysis revealed a statistically significant difference between the general level of knowledge—after classes, students achieved significantly better scores. This study highlighted changes in students’ awareness and knowledge about cancer brought about by implementing a cancer education project. Generally, high school students in Poland have basic knowledge about cancer and its risk factors. Most adolescents are aware of the significant impact of lifestyle on cancer risk. Early education on cancer prevention, mainly aimed at lifestyle changes, is crucial. Enhancing adolescents’ understanding of risk factors can play a significant role in lowering future cancer rates.

**Supplementary Information:**

The online version contains supplementary material available at 10.1007/s13187-025-02604-3.

## Introduction

With an estimated 20 million new cases and 9.7 million deaths in 2022, cancer is the second most common cause of death worldwide and a genuine global health problem. At the same time, The Polish National Cancer Registry reported about 181,300 new cases and 96,062 cancer deaths in 2022, also placing cancer as the second leading cause of death in Poland. It is estimated that in the next few dozen years, it will become the most common cause of death, thus ahead of cardiovascular disease. The global lifetime risk of cancer is estimated to be one in four for both men and women. However, the estimated lifetime risk of being diagnosed with cancer is almost 1 in 2 in developed countries, achieving 45% for females and 50% for males, respectively [1]. The World Health Organization projects that global new cancer cases will reach 21.6 million by 2030, with cancer-related deaths exceeding 12 million. Additionally, cancer imposes a significant clinical burden, disrupts social norms, and consumes extensive economic resources. This disease can impact people of any age, gender, or race [2].

The idea of health promotion grew out of the 1974 Lalonde report. Lalonde’s report introduced the concept of the “health field,” which comprises four interdependent areas that impact an individual’s health: lifestyle, environmental factors, biology, and healthcare organization [3]. Approximately half of cancer cases are linked solely to behavioral habits. Modifying behaviors can have a substantial impact on the rate of cancer cases. Lifestyle changes made at a young age are easier to adopt and have lasting effects on future health, making early awareness about cancer essential. A healthy lifestyle and avoiding cancer risk factors are achievable when individuals understand how their actions today will influence their long-term health [4]. However, there is little evidence on the long-term effectiveness of cancer education for high school students [5].

Research shows that teenagers lack adequate awareness of cancer risk factors. Recent studies indicate that a large number of young people lack knowledge about cancer prevention and often engage in behaviors that increase cancer risk, such as smoking and tobacco use, excessive alcohol consumption, physical inactivity, and poor diet [6]. Awareness of risk factors and cancer prevention principles is a key element in the fight against the twenty-first century epidemic that cancer has become. Studies indicate that early interventions can lessen the long-term effects of unhealthy behaviors developed during adolescence. Educating young people about cancer prevention is a vital step toward reducing the number of cancer-related deaths annually [7]. Adolescence is a key period for cancer risk education and intervention, as it is a time of active learning and a stage when risky behaviors, such as smoking, often begin. Research shows that adolescents are often not well-informed about the link between lifestyle choices and cancer risk [8]. Thus, recognizing personal risk factors and promoting early cancer prevention are essential. Prevention is a tool that allows for the reduction of cancer incidence, morbidity, and mortality. The World Health Organization recommends intensifying efforts to reduce the number of cases and deaths from malignant cancers, and this recommendation inspired the creation of the “OncoAcademy: The Key to Health” project, aimed at high school Polish students. The project was conducted in high schools to promote a positive health attitude and increase awareness among young people about cancer and methods of its prevention. The aim of the study is to analyze the attitudes and level of knowledge of Polish youth regarding cancer prevention and to evaluate the effectiveness of the educational activities conducted in this area.

## Methods

The program was designed for high school students as they are young individuals who, at this stage of their education, should have access to substantive knowledge about cancer, which is considered a lifestyle-related disease. Young people should understand the causes of cancer, the possibilities for early detection, and the importance of prevention as an effective tool in the fight against cancer. Gaining comprehensive knowledge about cancer at a young age can significantly influence these students in adulthood, enabling them to make informed health decisions. With this knowledge acquired early in life, they will be better equipped to prevent health and life-threatening risks as adults. Education of this nature, introduced at this stage in life, is expected to foster a more cancer-aware society in the future, potentially resulting in fewer cases of advanced-stage cancers and a reduction in mortality.

The study was conducted as a part of the health promotion educational project “OncoAcademy: The Key to Health” provided by academic teachers from the Department of Oncology Propaedeutics, Medical University of Warsaw, Poland. This project focused on cancer education to enhance high school students’ understanding of cancer risk factors. Special lessons were prepared on the epidemiological and etiological aspects of cancer. The lesson content was created through collaboration between oncology researchers and clinical oncologists and was conducted by academic teachers. At the time of study (2023–2024), high school education in Poland consisted of 4 years. The project, along with the knowledge assessment, was conducted across all grade levels. The activities conducted as part of the project included delivering lectures to groups of 50 participants and two interactive seminars in smaller, 20-person groups at each school. Participation in the project for each student thus involved completing a test twice, attending a 3-h lecture, and participating in two 2-h seminars.

The lecture topics covered issues related to carcinogenesis, the epidemiology of malignant tumors in Poland and worldwide, environmental causes of diseases, and primary prevention. The European Code Against Cancer was also explained, along with the genetic basis of cancer, including the principles of inheritance of cancer predispositions.

The interactive seminar sessions focused on detailed topics related to selected cancers, such as breast cancer, cervical cancer, lung cancer, colorectal cancer, and malignant testicular tumors. The principles of prevention and early detection of cancer were discussed using educational boards and models. The most common symptoms associated with each type of cancer were also presented.

All public general high schools of Mazovian Voivodeship were invited to participate in our project. Information about the project was sent to all high schools in the region. Posters promoting the project were also published on the official website of the University and the Department, along with guidelines on how schools could apply for participation. Due to the vast area covered by the project and the fact that the Mazovian Voivodeship is the largest province in Poland (approximately 35,559 km^2^), the highest interest in participation came from schools located in Warsaw or within several dozen kilometers from the capital. According to data from the Central Statistical Office, during the project implementation period, there were 289 public high schools operating in the Mazovian Voivodeship. In accordance with the project’s guidelines, participation was determined on a first-come, first-served basis. The first 30 applications submitted by school principals were accepted for participation in the project. Ultimately, thirty high schools participated in the OncoAcademy program, which accounts for 10.38% of all high schools in the region. Around 100 students from each school attended the workshops, thus a total of 3000 students participated in the project. Among the first thirty schools to apply, there were 19 high schools from the capital city of Warsaw and 11 from municipalities neighboring the capital. All the schools were located in urban areas: 19 schools (63.33%) from Warsaw (population over 100,000), 7 schools (23.33%) from medium-sized cities (20,000–100,000 inhabitants), and 4 (13.33%) from small towns (less than 20,000 inhabitants). The schools participating in the project were considered equivalent, as all of them were general secondary schools (vocational schools, branch schools, and technical schools did not participate in the project).

From September 2023 to November 2024, a total of 3000 students were surveyed, comprising 2195 girls and 805 boys (13–19 years old) in high schools in the Masovian Voivodeship, Poland. The survey was conducted twice in each class, 1 week before the educational project (pre-test) and another at the end of the class (post-test). Of the study participants who attended the cancer class, complete responses were obtained from 3000 students. A custom-designed questionnaire was utilized for this study (Supplementary Materials). It included 26 primary closed-ended single-choice quantitative questions, along with 2 questions collecting demographic information (age, gender) and the school name. The questions concerned basic issues related to cancer prevention, cancer epidemiology, risk factors, and carcinogens. The questionnaire contents were the same at both time points (i.e., pre-test and post-test). An analysis of the representativeness of the test questions was conducted during the preparation stage by comparing the content of the questions with the core curriculum, the teaching program, and the educational project program. It was verified whether the questions covered all key topics in appropriate proportions and did not omit essential aspects of knowledge. A psycho-oncologist confirmed its clarity and suitability. Participation in the study was both voluntary and anonymous.

Participation in the project was voluntary and open to every student from the registered school. However, there was a limit of about 100 applications from each school due to the group size restrictions at each stage of the project (lectures, interactive seminars, workshops). School principals or designated teachers were responsible for collecting student applications. According to the available data submitted by the registered schools, the total number of students in these institutions during this period was 20,097. The smallest school had 361 students, while the largest had 789 students. This shows that, on average, 14.93% of the students in the registered schools were surveyed (range 12.67–27.70%). There were no cases of refusal to participate in the project, as it was based on voluntary participation and registration.

The research was carried out with the consent of the students and/or students’ parents or legal representatives of the participants and the school principal. We verbally informed all participants that their involvement in the study was voluntary and that the collected data would be used exclusively for the purposes of this research. Furthermore, by submitting the questionnaire, the participants provided their informed consent.

In compliance with Polish regulations, IRB approval was not necessary for our study.

In the first stage of the analysis, the distributions of responses to individual questions from the questionnaire assessing students’ knowledge before and after the lessons were examined. Additionally, it was verified whether changes in knowledge levels were associated with gender, age, and school. For this purpose, frequency analysis was first performed for each question, comparing results before and after the lessons. Then, the McNemar test was used to compare the frequency of correct answers before and after the lessons, and chi-square independence tests were applied to compare students differentiated by gender, age, and school in terms of changes in knowledge levels within the studied area.

In the second stage of the analysis, the overall level of knowledge of the students before and after the lessons was assessed, and it was verified whether the change in knowledge was related to gender, age, and school. To do this, knowledge level indicators were first calculated. These included the sum of all correct answers (quantitative score) and the ratio of the sum of correct answers to the maximum possible score (percentage score). Next, basic descriptive statistics were calculated for the results, including measures of central tendency (mean, median), measures of dispersion (standard deviation, minimum, and maximum), measures of distribution shape (skewness and kurtosis), and the result of the Shapiro-Wilk test for normality of distributions. Subsequently, the Student’s t-test for dependent samples was used to compare the results before and after the lessons, the Mann-Whitney test was applied to compare two unequal groups in terms of the change in knowledge level, Pearson’s r correlation analysis was used to examine the linear relationship between age and the change in knowledge, and the Kruskal-Wallis test was applied to compare more than two unequal groups in terms of the change in knowledge level.

In the final step of the analysis, it was examined whether the school attended by the students influenced the change in their knowledge level. To do this, a one-way analysis of variance was used to compare students from different schools in terms of the level of the subject variable.

Statistical analysis was performed using Statistica v.13.3 (StatSoft).

## Results

The study was conducted on 3000 students (aged 13–18) from 30 high schools in Mazovian Voivodeship, Poland. The majority of them (19) were schools from Warsaw. Then, 2195 girls and 805 boys participated in the study. The disproportion in the participation of boys and girls in the project results from the fact that it was directed to willing students based on voluntary registration. The total number of students in the participating schools was 20,097, including 12,378 girls (61.59%) and 7719 boys (38.41%). These data indicate, on one hand, the ongoing trend of a higher number of female students in Polish general high schools and, on the other hand, a greater interest among girls in the project’s subject matter. The participation rates were thus 17.73% for girls and 10.43% for boys, respectively. The age of project participants was 13–19 years (average 15.6 years; SD ± 1.11).

In the first stage of the analysis, the distributions of responses to individual questions from the questionnaire assessing students’ knowledge before and after the lessons were examined. Table 1 provides cross-sectional results and the most frequently chosen and correct answers followed by the percentage of students who improved/worsened their scores after participating in the classes. Table 2 presents the number of correct answers for individual questions during the pre-test (before the project) and the post-test (after the project), as well as the change in the level of knowledge regarding specific topics.

**Table 1:** Cross-sectional results in responses to individual questions

No	Question	The most frequently chosen answer	Correct answer	Improvement OR deterioration in score	The percentage of students who improved/worsened their score	*p*-value
1	How many cases of malignant cancers are recorded annually in Poland?	180,000		Deterioration	28%	** < **0.001
2	Which type of cancer is most commonly diagnosed in women?	Breast cancer		Improvement	25.4%	** < **0.001
3	Which type of cancer causes the most deaths in Poland?	Lung cancer		Improvement	42.2%	** < **0.001
4	Which virus contributes to the development of cervical cancer?	HPV		Improvement	24.6%	** < **0.001
5	Which factor has the most significant impact on the occurrence of lung cancer?	Smoking cigarettes		Improvement	13.0%	** < **0.001
6	Which test is not considered a screening test for early cancer detection?	Chest X-ray		Improvement	29.8%	** < **0.001
7	What is the percentage of curability in oncology?	30% and 50%	50%	Improvement	22.7%	** < **0.001
8	Which factor does not have carcinogenic potential?	Ultrasound		Improvement	21.8%	** < **0.001
9	Which examination is essential for diagnosing malignant tumors?	Histo-pathological examination and imaging examination	Histo-pathological examination	Improvement	23.6%	** < **0.001
10	When can a hereditary basis for cancer be suspected?	All correct (develops at a younger age than in the general population, occurs in several close relatives, occurs in at least two generations)		No difference	20.6% better 18.9% worse	0.147
11	What is the sequence of carcinogenesis stages?	Initiation–progression–promotion and initiation–promotion–progression	Initiation–promotion–progression	Improvement	30.8%	** < **0.001
12	At what age is mammography recommended for women?	30–59 and 40–59	50–69	Improvement	25.2%	** < **0.001
13	Which factor is not a risk factor for breast cancer?	Breastfeeding	Breastfeeding and smoking cigarettes	Deterioration	21.2%	** < **0.001
14	What is not a characteristic of malignant tumors?	The presence of a capsule surrounding the tumor		Improvement	32.0%	** < **0.001
15	Which marker is detected in blood serum for ovarian cancer diagnosis?	CA125		No difference	22.2% better 22.0% worse	0.869
16	What can be a symptom of lung cancer?	All correct (hemoptysis, shortness of breath, cough)		Improvement	11.9%	0.029
17	Which cancer is the leading cause of death among women in Poland?	Breast cancer and lung cancer	Lung cancer	Improvement	53.2%	** < **0.001
18	What is a risk factor for lung cancer?	All correct (use of electronic cigarettes, exposure to asbestos, ionizing radiation)		Improvement	22.8%	** < **0.001
19	How many years shorter is the life expectancy of people who smoke cigarettes or other tobacco products compared to non-smokers?	On average, 10 years		Improvement	36.3%	** < **0.001
20	What is not a risk factor for colorectal cancer?	A diet rich in fiber and physical activity		Improvement	24.9%	** < **0.001
21	Which HPV type has been shown to have the highest oncogenic potential for cervical cancer development?	HPV 16		Improvement	36.5%	** < **0.001
22	What is the most effective method of protection against HPV infection?	Vaccination		Improvement	28.7%	** < **0.001
23	What is the most common cancer of the male reproductive system?	Prostate cancer		Improvement	26.9%	** < **0.001
24	How often should women perform breast self-examinations?	Once in month		Improvement	37.0%	** < **0.001
25	How often is mammography recommended in breast cancer screening?	Every 2 years		Improvement	32.7%	** < **0.001
26	Which cancers are BRCA gene mutations associated with?	Breast cancer and ovarian cancer		No difference	25.8% better 23.9% worse	0.133

**Table 2:** Change in the level of knowledge regarding specific questions

No	Question	Number of correct answers (%) Pre-test	Number of correct answers (%) Post-test	Change in the knowledge level (%)
1	How many cases of malignant cancers are recorded annually in Poland?	1451 (48.4%)	1245 (41.5%)	− 6.9%
2	Which type of cancer is most commonly diagnosed in women?	2157 (71.9%)	2700 (90.0%)	18.1%
3	Which type of cancer causes the most deaths in Poland?	1572 (52.4%)	2667 (88.9%)	36.5%
4	Which virus contributes to the development of cervical cancer?	2122 (70.7%)	2486 (82.9%)	12.2%
5	Which factor has the most significant impact on the occurrence of lung cancer?	2571 (85.7%)	2794 (93.1%)	7.4%
6	Which test is not considered a screening test for early cancer detection?	1060 (35.3%)	1360 (45.3%)	10.0%
7	What is the percentage of curability in oncology?	804 (26.8%)	920 (30.7%)	3.9%
8	Which factor does not have carcinogenic potential?	2163 (72.1%)	2341 (78.0%)	5.9%
9	Which examination is essential for diagnosing malignant tumors?	852 (28.4%)	990 (33.0%)	4.6%
10	When can a hereditary basis for cancer be suspected?	2143 (71.4%)	2194 (73.1%)	1.7%
11	What is the sequence of carcinogenesis stages?	1285 (42.8%)	1615 (53.8%)	11.0%
12	At what age is mammography recommended for women?	446 (14.9%)	883 (29.4%)	14.5%
13	Which factor is not a risk factor for breast cancer?	799 (26.6%)	626 (20.9%)	− 5.7%
14	What is not a characteristic of malignant tumors?	1251 (41.7%)	1675 (55.8%)	14.1%
15	Which marker is detected in blood serum for ovarian cancer diagnosis?	1047 (34.9%)	1054 (35.1%)	0.2%
16	What can be a symptom of lung cancer?	2595 (86.5%)	2652 (88.4%)	1.9%
17	Which cancer is the leading cause of death among women in Poland?	566 (18.9%)	1987 (66.2%)	47.3%
18	What is a risk factor for lung cancer?	2143 (71.4%)	247 (80.6%)	9.2%
19	How many years shorter is the life expectancy of people who smoke cigarettes or other tobacco products compared to non-smokers?	1530 (51.0%)	2223 (74.1%)	23.1%
20	What is not a risk factor for colorectal cancer?	1965 (65.5%)	2171 (72.4%)	6.9%
21	Which HPV type has been shown to have the highest oncogenic potential for cervical cancer development?	1516 (50.5%)	2229 (74.3%)	23.8%
22	What is the most effective method of protection against HPV infection?	1870 (62.3%)	2279 (76.0%)	13.7%
23	What is the most common cancer of the male reproductive system?	1796 (59.9%)	2070 (69.0%)	9.1%
24	How often should women perform breast self-examinations?	1496 (49.9%)	2248 (74.9%)	25.0%
25	How often is mammography recommended in breast cancer screening?	1193 (39.8%)	1451 (48.4%)	8.6%
26	Which cancers are BRCA gene mutations associated with?	1570 (52.3%)	1629 (54.3%)	2.0%

Further, it was verified whether changes in knowledge levels to individual questions were associated with gender, age, and attending school. No significant results were achieved regarding age or sex, even in the questions solely concerning female cancers and preventive examinations (such as mammography screening or breast self-examination). On the other hand, in most questions, changes in knowledge levels were associated with attending school.

In the second stage of the analysis, the overall level of knowledge of the students before and after the lessons was assessed. The analysis revealed a statistically significant difference between the general level of knowledge—after classes, students achieved significantly better scores (Table 3).

**Table 3:** Comparison of the overall knowledge level index before and after the classes

Dependent variable	Before classes		After classes				95% CI	
	*M*	*SD*	*M*	*SD*	*t*	*p*	*LL*	*UL*
Level of knowledge—percentage results	0.52	0.14	0.62	0.14	− 29.56	** < **0.001	− 0.11	− 0.10

Abbreviations: *M*, mean; *SD*, standard deviation; *t*, test statistic value; *df*, degrees of freedom; *p*, statistical significance; *95% CI*, 95% confidence interval; *LL and UL*, lower and upper limits of the confidence interval

Results revealed that students’ level of knowledge before classes was moderate (*M* = 51.76%; SD = 13.63%). On average, students correctly answered 13 of 26 questions. After classes, the level of knowledge was assessed as moderately good (*M* = 62.47%; SD = 14.48%). On average, students correctly answered 16 out of 26 questions (Table 4). The strong knowledge of cancer awareness in the pre-test can be explained by the fact that the students who applied for the project were the most interested in the topic of cancer prevention and already had some knowledge in this area.

**Table 4:** Descriptive statistics of the studied variables along with the Shapiro-Wilk test

Variable	*M*	*Mdn*	*SD*	*Skew*	*Κ*	*Min*	*Max*	*S-W*	*p*
Before classes									
Quantitative result	13.46	13.00	3.54	0.29	0.17	2.00	24.00	0.99	** < **0.001
Percentage result	0.52	0.50	0.14	0.29	0.17	0.08	0.92	0.99	** < **0.001
After classes									
Quantitative result	16.24	17.00	3.76	− 0.89	1.20	1.00	25.00	0.95	** < **0.001
Percentage result	0.62	0.65	0.14	− 0.89	1.20	0.04	0.96	0.95	** < **0.001
Change in knowledge level									
Quantitative result	2.78	3.00	5.16	− 0.43	0.18	− 15.00	17.00	0.99	** < **0.001
Percentage result	0.11	0.12	0.20	− 0.43	0.18	− 0.58	0.65	0.99	** < **0.001

Abbreviations: *M*, mean; *Mdn*, median; *SD*, standard deviation; *Skew.*, skewness; *Κ*, kurtosis; *Min.*, minimum; *Max.*, maximum;* S-W*, Shapiro-Wilk test results; *p*, *p*-value for Shapiro-Wilk test

**Fig. 1:**
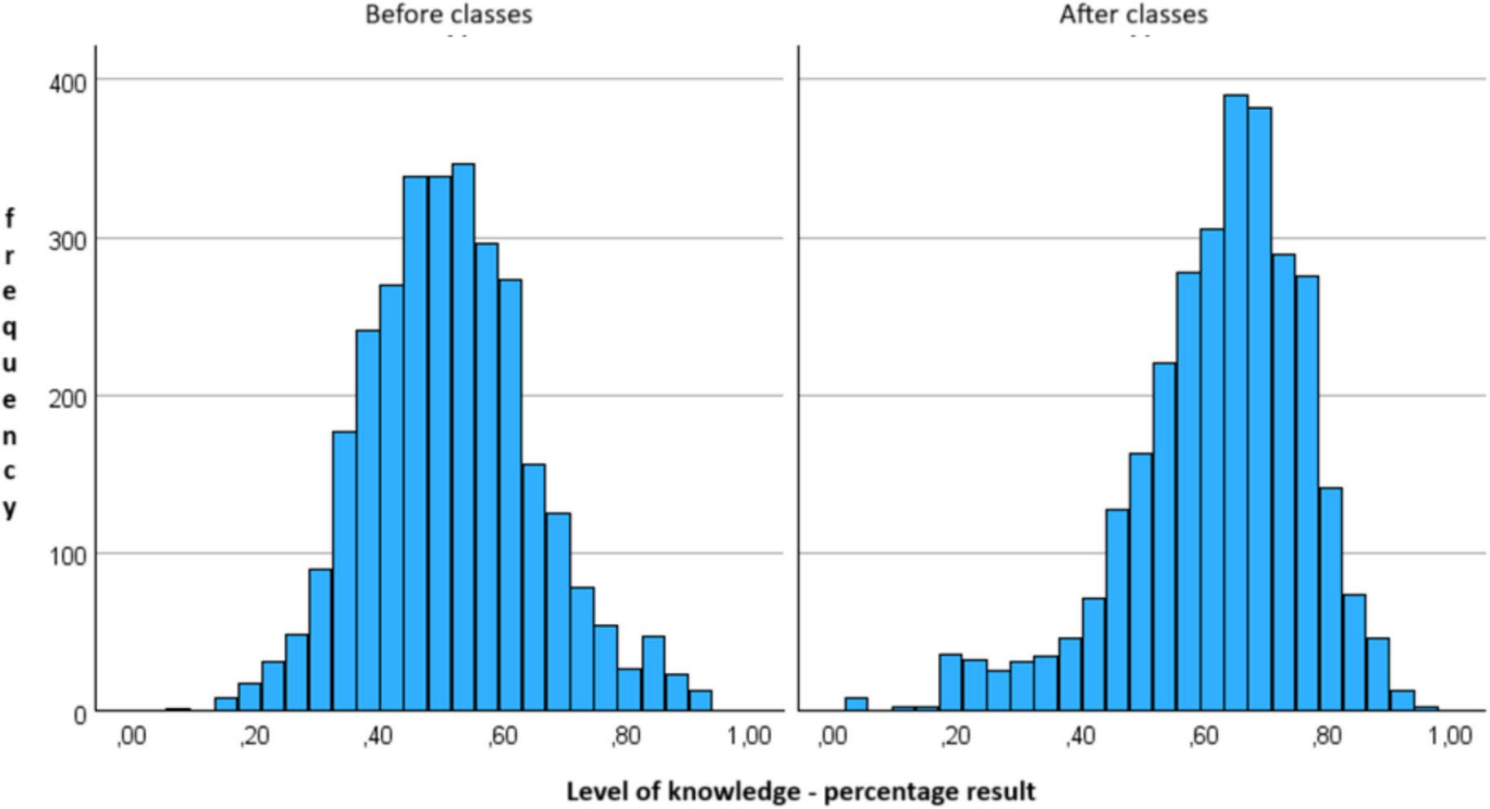
Histograms of the percentage index of overall knowledge level before and after the classes

Moreover, before classes, the positive value of skewness suggested that most of the achieved results were below average. On the contrary, the skewness value was negative after classes, meaning that most achieved results were above average (Fig. 1).

In the context of gender, the study revealed no difference in knowledge between female and male participants. Similarly, no difference was observed between younger and older students. Notably, a statistically significant difference was calculated between different schools (*p* < 0.001). The change in knowledge levels across the schools was assessed using a one-way analysis of variance (post hoc T2 Tamhane test). The result reflects the average difference in scores between the pre-test and post-test in each school. The obtained results indicate that students from schools who scored lower in the pre-test showed a significantly greater change in knowledge levels in the post-test, compared to students from schools with a high baseline level of knowledge.

## Discussion

Establishing healthy habits during adolescence is crucial for preserving good health into adulthood. Some researchers have pointed out that high school and university students often have limited knowledge about cancer risk factors. Understanding these risk factors provides an opportunity to avoid them, thereby reducing the risk of developing cancer. In Poland, there is a lack of research on the attitudes and awareness of cancer risk factors and prevention among high school students. Only the study by Rucińska et al. and Moskal et al. assessed students’ knowledge in this area, although it focused only on a few selected aspects [9, 10].

According to the annual report of the National Cancer Registry, cancers were the second cause of death in Poland, accounting for 18.8% of deaths among men and 17.2% among women. Malignant tumors represent a significant health issue, particularly among young and middle-aged individuals (aged 15–64). The most prevalent male cancer is prostate cancer, followed by lung cancer. Nevertheless, for the last 20 years, we have witnessed a reduced incidence of lung cancer among the male population—new cases among men have decreased by 22% since 2000, which is directly associated with a reduced number of smokers. In the female population, the most common cancer is breast cancer, followed by lung and colorectal malignancies [11].

In the present study, we conducted a cancer education class for high school students and assessed the changes in their awareness and knowledge about cancer. Our findings revealed significant improvements following the educational project. Participants identified various risk factors contributing to cancer development, including behavioral, physiological, and environmental aspects. They acknowledged individual risks such as sun exposure, smoking or tobacco use, diet, alcohol consumption, and drug use as contributors to cancer. However, some participants admitted to lacking knowledge about cancer prevention. According to the World Health Organization (WHO), approximately 30–50% of cancer deaths could be prevented by avoiding major risk factors, coupled with early detection and diagnosis. Awareness of cancer signs and symptoms plays a crucial role in reducing mortality by enabling early diagnosis and treatment. Educational interventions targeting adolescents can enhance their understanding of cancer prevention and potentially lead to long-term benefits, including improved survival rates [12, 13].

Tobacco smoking is a significant risk factor for developing various cancer types, including lung cancer. It is estimated that 80–90% of lung cancer cases are associated with cigarettes [14, 15]. In the context of lung cancer, our seminar sessions and further survey questions focused on risk factors (especially cigarette smoking), symptoms, and early detection. In our observation during seminars, students were aware of the influence of smoking on lung or pharynx malignancies; however, they were somewhat surprised about the influence of tobacco on other cancers (such as cervical or pancreatic cancers) or cardiovascular diseases. Our observations align with results from the national Polish study from 2019. The study participants were a random sample of 1000 individuals, representative of the general population of Poland aged 15 and older. One of the main conclusions from the study was that Poles are relatively consistent that smoking causes lung cancer, while to a much lesser extent, agree that smoking is associated with strokes or heart attacks [16]. In 2015, a survey about the influence of smoking on lung carcinoma was carried out among high school students in Poland. Interestingly, in the analyzed group, 84.2% of respondents chose smoking as the leading lung cancer risk factor, but concurrently, 67.3% were active smokers. Additionally, smokers presented a lower level of knowledge about lung cancer in comparison to non-smokers [17]. In conclusion, more effort should be put into education about the various risks associated with smoking cigarettes. The risk of developing lung cancer itself seems insufficient to discourage smoking.

The cancer diagnosis stage is the most significant variable for further treatment response and prognosis. Society’s awareness of early symptoms and screening examinations might lead to diagnosis in earlier stages and result in a better prognosis. The most prevalent symptoms of lung cancer encompass shortness of breath, fatigue, coughing (also hemoptysis), chest pain, and weight loss [18]. In our study, almost 90% of students correctly chose symptoms of lung cancer—hemoptysis, shortness of breath, and cough. In 2023, the analysis focusing strictly on lung cancer symptoms was conducted among pharmacy and nursing undergraduates in Saudi Arabia. The results were far more disappointing, as only about 40% of participants reported coughing up blood and worsening or changing an existing cough as symptoms of lung cancer [19]. In the study by Alrabeeah et al. conducted on over 15,000 participants, 60% of respondents showed low confidence in identifying the signs and symptoms of lung cancer [20]. Awareness of the risk factors and symptoms of lung cancer depended on age, gender, education, marital, and employment status.

As previously mentioned, breast cancer is the most prevalent malignancy among women in Poland. Other gynecological cancers, such as endometrial, cervical, and ovarian, are also in the first ten of the most commonly diagnosed malignancies. They are also among the first ten most deadly cancers [11]. The statistics have been quite stable over the past few years despite implementing prophylaxis programs for breast and cervical cancers. Our study proved that the knowledge about prophylaxis programs is quite good; nevertheless, according to official national statistics from 2024, only about 30% of qualified Polish women benefit from mammography and only ~ 11% from cytology [21]. Results of the systematic review of 35 studies suggest that most women are aware of mammograms; however, there was significant variability regarding the awareness of the purpose of screening [22]. In the Polish study, among 100 women aged 20–45, almost 100% knew about the existence of mammography, but details about the frequency or the purpose of the examination were known by only 30–50% of respondents [23]. In various studies, the barriers most encountered towards mammography encompass a lack of detailed information about mammograms, fear of radiation exposure, fear of error in diagnosis, and fear associated with the possible discovery of cancer and further oncological treatment [24, 25]. Social campaigns and lessons for students about the details of prophylaxis programs and examinations are highly needed to overcome barriers leading to low attendance to cytology and mammography.

Given the current low rates of participation in cancer screening tests, we created opportunities for students to independently and collaboratively explore and present the reasons for undergoing such screenings. We believe these learning activities enhanced students’ understanding of the importance of regular checkups and the potential to overcome cancer through early detection and timely treatment.

A significant improvement was noted after the classes regarding young people’s knowledge of breast cancer prevention. After the classes, 37% more young people knew that every woman over the age of 20 should perform a breast self-examination once a month. It is essential because, according to the National Cancer Registry in Poland, among young adults (20–44 years old), standardized cancer incidence rates are twice as high as among men. Among middle-aged women, cancer causes over 1/3 of deaths (35% of deaths in 2021). In Poland, in the group of women aged 20 to 44, the most common cancer in terms of incidence is breast cancer. That is why it is so crucial for young people to know what breast cancer prevention is, to be able to reduce the risk of developing this cancer in the following years of life [11, 26].

More and more children in Poland are diagnosed with overweight and obesity. According to the World Health Organization (WHO), overweight and obesity were recorded in 32% of Polish children aged 7–9—8th place among European countries studied [27, 28]. Therefore, greater knowledge of children and adolescents on obesity prevention and the impact of this obesity on the development of many cancer diseases is crucial.

A recent meta-analysis by Shivappa et al. demonstrated increased risk in the incidence of CRC with certain foods by using the Dietary Inflammatory Index of food [29]. A higher DII score correlated with the pro-inflammatory potential, thereby increasing the risk of CRC. In contrast, a lower DII score correlated with anti-inflammatory potential, reducing the risk of CRC. In our study, approximately 25% of students, after the educational project, knew that dietary fiber and physical activity do not increase the risk of developing colon cancer. The knowledge that students gain may, in the future, translate into changing their habits and lifestyle to a more health-promoting one and to a lower risk of developing cancer.

A higher level of knowledge among high school students on cancer prevention after completing the project may translate into passing on this knowledge to their families and friends. The knowledge gained may influence the change in eating habits, where we know that improper diet is responsible for approximately 30% of cancer cases. Excessive body weight increases the risk of developing as many as 11 malignant tumors (including colon, kidney, and gallbladder cancer) [30].

A limitation of this study lies in its sample, which consisted solely of high school students from a single voivodeship in Poland. Additionally, the absence of a control group prevents immediate generalization of the findings.

This study highlighted changes in students’ awareness and knowledge about cancer brought about by implementing a cancer education project. Generally, high school students in Poland have basic knowledge about cancer and its risk factors. Most adolescents are aware of the significant impact of lifestyle on cancer risk. Our findings revealed that the proposed cancer education class led to short-term improvements in junior high school students’ awareness and understanding of cancer. It will be extremely valuable to assess the level of knowledge after a longer period following the educational sessions, as this will allow for an evaluation of the long-term effects of the project and the drawing of conclusions regarding the effectiveness of such initiatives. Early education on cancer prevention, mainly aimed at lifestyle changes, is crucial. Enhancing adolescents’ understanding of risk factors can play a significant role in lowering future cancer rates.

## Supplementary Information

Below is the link to the electronic supplementary material.

Supplementary file1 (DOCX 23 KB)

## Data Availability

The data presented in this study are available on request from the corresponding author.
