# Supplementary material for: The Impact of an Educational Project on Cancer-Related Knowledge and Awareness Among High School Students
Source: J Cancer Educ. 2025 Mar 18;40(6):947–57. doi: 10.1007/s13187-025-02604-3 (PMC12717195; doi:10.1007/s13187-025-02604-3)
Supplement: Supplementary file 1 — Supplementary file1 (DOCX 23 KB) [file 13187_2025_2604_MOESM1_ESM.docx]

**OncoAcademy: The Key to Health**

Participant Information [Please fill in or select the appropriate options]

Age: .....................................................................................................................

Gender: .............................................................................................................

Type of School: General High School / Technical School / Other ................................................

Class Profile: ......................................................................................................

Questions

1. How many cases of malignant cancers are reported annually in Poland?

a. 50,000

b. 100,000

c. 180,000

d. 250,000

2. Which cancer is most commonly diagnosed in women?

a. Uterine cancer

b. Breast cancer

c. Colorectal cancer

d. Cervical cancer

3. Which cancer causes the highest number of deaths?

a. Lung cancer

b. Colorectal cancer

c. Stomach cancer

d. Brain tumours

4. Which virus contributes to the development of cervical cancer?

a. HIV

b. VZV

c. HSV

d. HPV

5. Which factor has the greatest influence on the development of lung cancer?

a. Ionizing radiation

b. Smoking cigarettes

c. Environmental pollution

d. Genetic factors

6. **Screening tests** performed for early cancer detection include all of the following **except**:

a. Chest X-ray

b. Pap smear

c. Mammography

d. Colonoscopy

7. The cure rate in oncology is approximately:

a. 10%

b. 30%

c. 50%

d. 70%

8. Which of the following is **NOT** a carcinogenic factor?

a. UV radiation

b. By-products of fuel combustion

c. Tobacco smoke

d. Ultrasound

9. A necessary test for diagnosing malignant cancer is:

a. Histopathological examination

b. Medical examination by a doctor

c. Tumour marker testing in the blood

d. Imaging tests, e.g., CT scan

10. Hereditary cancers may be suspected when:

a. Cancer develops at a younger age than in the general population

b. Cancer occurs in several close relatives

c. Cancer appears in at least two generations

d. All of the above are correct

11. Indicate the correct sequence of carcinogenesis stages:

a. Promotion – Progression – Initiation

b. Initiation – Promotion – Progression

c. Initiation – Progression – Promotion

d. Promotion – Initiation – Progression

12. Mammographic screening is recommended for women:

a. Between the ages of 30-59

b. Between the ages of 40-59

c. Between the ages of 50-69

d. Between the ages of 60-69

13. Which of the following is **NOT** a risk factor for breast cancer?

a. Breastfeeding

b. Excessive alcohol consumption

c. Smoking

d. Both a and c

14. Which is **NOT** a characteristic of malignant tumours?

a. High growth rate

b. Presence of a capsule surrounding the tumour

c. High mitotic index

d. Presence of metastases

15. In the diagnosis of ovarian cancer, blood serum is tested for the level of:

a. ACTH

b. CA 125

c. GH

d. PSA

16. A symptom of lung cancer may be:

a. Haemoptysis

b. Cough

c. Shortness of breath

d. All of the above are correct

17. Which cancer is the leading cause of cancer deaths among women in Poland?

a. Lung cancer

b. Breast cancer

c. Ovarian cancer

d. Pancreatic cancer

18. Risk factors for lung cancer include all of the following **except**:

a. Using electronic cigarettes

b. Asbestos exposure

c. Ionizing radiation

d. All of the above are risk factors for lung cancer

19. On average, how many years shorter is the lifespan of smokers compared to non-smokers?

a. 5 years

b. 10 years

c. 15 years

d. 20 years

20. Which is **NOT** a risk factor for colorectal cancer?

a. A diet high in fiber

b. A diet high in meat

c. Physical activity

d. Both a and c

21. The highest oncogenic impact on the development of cervical cancer has been shown for:

a. HPV 11

b. HPV 16

c. HPV 31

d. HPV 45

22. The most effective method of protecting against HPV infection is:

a. Vaccination

b. Using hormonal contraception

c. Frequent Pap smears

d. Eating a diet rich in antioxidants

23. The most common cancer of the male reproductive system is:

a. Testicular cancer

b. Penile cancer

c. Prostate cancer

d. Vas deferens cancer

24. Every woman should perform breast self-examinations:

a. Once a week

b. Once a month

c. Once a quarter

d. Once a year

25. Mammographic screening for breast cancer is recommended:

a. Annually

b. Every 2 years

c. Every 3 years

d. Every 5 years

26. Mutations in the BRCA gene predispose individuals to the development of:

a. Breast cancer

b. Ovarian cancer

c. Lung cancer

d. Both a and b
